# Perfluorinated chemicals and adolescent respiratory health: Epidemiological evidence and mechanistic insights

**DOI:** 10.1371/journal.pone.0336788

**Published:** 2025-11-14

**Authors:** Xinfeng Xu, Xinyao Jiang, Meng Zou, Jinyan Hui, Guang Huang, Qian Wu

**Affiliations:** China International Cooperation Center (CCC) for Environment and Human Health and Department of Health Inspection and Quarantine, School of Public Health, Nanjing Medical University, Nanjing, China; University of South Carolina, UNITED STATES OF AMERICA

## Abstract

Perfluorinated compounds (PFCs) are persistent environmental pollutants with near-universal human exposure, yet their respiratory health impacts during adolescence remain insufficiently explored. This investigation evaluated single and combined effects of serum PFCs on pulmonary function and respiratory morbidity in a nationally representative adolescent cohort (n = 976, ages 12–19 years) utilizing 2007–2012 NHANES data. Advanced analytical approaches including multivariable regression, mixture modeling (BKMR and WQS), and mediation analysis were employed to assess associations with spirometric parameters (FEV_1_, FVC, FEV_1_/FVC) and respiratory symptoms while examining inflammatory and oxidative stress pathways. Computational approaches integrating network toxicology and molecular docking identified key protein targets. Analytical results demonstrated significant associations between specific PFC congeners (PFOA, PFHS, PFOS) and pulmonary function measures, with age-stratified effects observed for wheezing symptoms. Mixture analyses revealed PFOA as the predominant contributor to observed respiratory effects, partially mediated through oxidative stress pathways (6.8–8.2% mediation). Molecular investigations identified critical signaling nodes (INS, AKT1, TP53, TNF, IL6, ALB and PPARγ) potentially linking PFC exposure to respiratory outcomes. These findings provide mechanistic insights into PFC-induced pulmonary effects during adolescence, highlighting the need for continued investigation of these environmentally persistent compounds’ impact on developing respiratory systems. The integrated epidemiological-computational approach demonstrates the utility of combining population-level data with mechanistic modeling to elucidate environmental health effects.

## 1. Introduction

Perﬂuorinated chemicals (PFCs) are synthetic compounds characterized by a fully ﬂuorinated carbon backbone of variable length, terminated by an ionic functional group (typically carboxylate or sulfonate) [1,2]. These compounds possess unique characteristics such as water and oil repellency, thermal stability, and chemical resistance, making them valuable for commercial and industrial applications [3–5]. The extraordinary stability of PFCs’ carbon-fluorine bonds renders them highly persistent environmental contaminants [6]. Global monitoring studies have detected these compounds across diverse environmental matrices [2,7,8]. Key long-chain variants – including perfluorooctanoic acid (PFOA), perfluorooctane sulfonic acid (PFOS), perfluorononanoic acid (PFNA), and perfluorohexane sulfonic acid (PFHS) – exhibit remarkable persistence in humans, with biological half-lives spanning several years [9–11]. Epidemiologic studies detect these compounds in serum samples from >98% of the U.S. population, including children [10,12–15]. PFCs, particularly PFOA and PFOS, are recognized as priority contaminants due to their multiple exposure pathways (ingestion, dermal absorption, and inhalation) and demonstrated toxic effects in animal studies, including hepatic, developmental, immunological, neurobehavioral, endocrine, and metabolic toxicity [10,16–19].

Recent epidemiological studies have shown that serum PFC exposure may contribute to respiratory dysfunction, including asthma. An early analysis of the National Health and Nutrition Examination Survey (NHANES) data from 1999–2000 and 2003–2008 cycles revealed a significant positive association between serum PFOA levels and asthma diagnosis among adolescents aged 12–19 years [20]. Another study found an association, especially for PFUA exposure, with asthma among 3–11-year children from the 2013–2014 [21]. However, a study by using linear regression and weighted quantile sum regression found no associations between PFOA, PFNA, PFHS and PFOS and mixtures and the pulmonary function measures in 12–19 years adolescents (NHANES 2007–2012) [22]. But in the 2009–2010 Genetic and Biomarkers study for Childhood Asthma, PFAS concentrations showed significant associations with impaired pulmonary function in children with asthma [23].

Children and adolescents undergo critical lung development with heightened vulnerability to environmental pollutants. Growing evidence indicates early-life contaminant exposure may impair pulmonary maturation and elevate the risk of respiratory diseases in adult [24]. However, the results of the effect of PFCs on respiration health in children and adolescents is inconsistent. Consequently, there is a critical need to precisely characterize the impact of serum PFCs on respiration outcomes, specially impaired lung function and asthma development in children and adolescents.

In this study, we aimed to explore the association between serum PFC levels and health indicators in 12–19 aged children and adolescents using data from NHANES, 2007–2012. To assess the combined effects of PFC exposure on respiratory health, we applied mixture-exposure models. Additionally, we employed network toxicology analyses to explore potential toxicity pathways and molecular mechanisms underlying PFC-induced lung function impairment. Our findings, derived from a broad range of PFC exposure levels, may enhance the understanding of PFC-related health risks in pediatric populations.

## 2. Materials and methods

### 2.1. Study population

This study analyzed the data from a representative adolescent cohort (n = 976, ages 12–19 years) utilizing 2007–2012 NHANES data, which were accessed on April 1, 2025. NHANES is a national survey that assesses the health and nutrition status of adults and children across the United States, which offers open-access data and documentation for public use. A total of 976 eligible participants aged 12–19 years with serum-PFC measurements were included from the 2007–2008, 2009–2010, and 2011–2012 NHANES cycles, and they have provided lung function indicators and responses to the questions regarding respiration health in the questionnaires. NHANES data are publicly available, de-identified data approved by the National Center for Health Statistics (NCHS) Research Ethics Review Board, with study protocol approved by this review board and written consent obtained from all surveyed individuals. In addition, NHANES data are disseminated in compliance with strict confidentiality and privacy protection standards. As such, the authors did not have access to privacy information that could identify individual participants at any stage.

### 2.2. Exposure assessment

Serum PFCs were measured using standardized NHANES laboratory protocols. Briefly, solid phase extraction coupled to High Performance Liquid Chromatography-Turbo Ion Spray ionization-tandem Mass Spectrometry (online SPE-HPLC-TIS-MS/MS) was employed for the quantitative detection. For quantification, the internal standard method was employed. The following serum PFCs were detected: perfluoroheptanoic acid (PFHP), perfluorooctanoic acid (PFOA), perfluorononanoic acid (PFNA), perfluorodecanoic acid (PFDE), perfluoroundecanoic acid (PFUA), perfluorododecanoic acid (PFDO), perfluorobutane sulfonic acid (PFBS), perfluorohexane sulfonic acid (PFHS), perfluorooctane sulfonic acid (PFOS), perfluorooctane sulfonamide (PFSA), 2-(N-ethyl-perfluorooctane sulfonamido) acetic acid (EPAH), and 2-(N-methyl-perfluorooctane sulfonamido) acetic acid (MPAH). Samples with non-detectable levels were assigned a value of LOD/√2 in accordance with standard practice.

### 2.3. Health indices

The spirometry data collection is designed to investigate the prevalence of asthma and adult chronic obstructive pulmonary disease (COPD) in the U.S. population. In this study, three key spirometric parameters were analyzed: forced vital capacity (FVC), forced expiratory volume in 1 second (FEV_1_), and their percentage ratio (FEV_1_/FVC%), with the latter serving as the primary diagnostic criterion for obstructive lung disorders in current NHANES spirometry study.

Data on wheezing manifestations and asthma diagnosis were collected through health questionnaires. Current wheezing was defined based on an affirmative response to experiencing chest wheezing or whistling sounds within the previous year. Asthma cases were identified based on self-reported professional medical diagnoses.

Complete blood cell counts, including lymphocytes, neutrophils, monocytes, and platelets, were quantified using the Beckman Coulter DXH 800 hematology analyzer. Inflammation biomarkers were calculated as follows: neutrophil/lymphocyte ratio (NLR), systemic immune-inflammation index (SII = platelets × neutrophils/lymphocytes), and systemic inflammation response index (SIRI = monocytes × neutrophils/lymphocytes). Serum concentrations of oxidative stress biomarkers-total bilirubin and gamma-glutamyl transferase (GGT)-were quantitatively analyzed [25,26].

### 2.4. The prediction of PFC-related target genes

The potential molecular target connecting PFC exposure to juvenile respiratory disorders were systematically identified through GeneCards database and CTD database. Subsequent bioinformatics characterization was conducted through DAVID-based Gene Ontology (GO) term classification and Kyoto Encyclopedia of Genes and Genomes (KEGG) pathway enrichment studies. The Cytoscape (Version 3.9.1) was employed to construct the protein-protein interaction network (PPI). Subsequently, hub targets were systematically identified by evaluating seven topological parameters: maximal clique centrality (MCC), maximum neighborhood component (MNC), edge percolated component (EPC), stress, degree, closeness, and betweenness.

Protein structures for key targets were sourced from the RCSB Protein Data Bank, while the PFC configuration was obtained through PubChem. Subsequently, PFC, designated as the ligand, and the hub proteins, regarded as the receptors, were imported into AutoDockTools version 1.5.6, involving the following steps: (1) removal of crystallographic water molecules, (2) addition of polar hydrogens, and (3) computation of partial atomic charges. The prediction of ligand binding pockets in the protein of interest was carried by Prankweb. Protein-ligand docking simulations was performed using AutoDock Vina, with subsequent visualization and interaction analysis in PyMOL.

### 2.5. Statistical analysis

The baseline characteristics, e.g., demographic and clinical characteristics in the population were evaluated using descriptive analytical approaches. Continuous variables were described as median with interquartile range (IQR), and categorical variables were presented as numbers (n) and frequency (%). Spearman correlation coefficients was used to assess the associations between eight serum PFCs.

Associations between serum PFC concentrations and respiratory health parameters were evaluated using multiple linear regression, with results presented as regression coefficients (beta) or odds ratios (OR) value and 95% confidence interval (CI). In order to investigate potential disparities attributed to gender and age, the associations between serum PFCs and lung health were separately analyzed across distinct age and gender subgroups. Furthermore, the dose-response patterns linking serum PFC exposure to respiratory outcomes (lung function, wheeze, and asthma) were further explored using restricted cubic spline (RCS) regression.

Generalized Linear Model was used to assess the relationship between oxidative stress and inflammation related index and lung health as well as serum PFCs. A mediation analysis was employed to assess the mediating role of inflammation-related indices in the association between serum PFCs and lung health.

Due to the uncertain functional relationships between the PFC exposure levels and pulmonary function parameters (FEV_1_, FVC, FEV_1_/FVC), as well as respiratory conditions (wheeze and asthma), we initially applied machine learning (ML) methods for variable selection. We used the “pycaret” to get the training accuracy of different ML classification models and R2 of different ML regression models. After identifying the best-performing model through accuracy and R^2^ metrics, we conducted additional training and utilized SHapley Additive exPlanation (SHAP) methodology to interpret variable contributions, while maintaining 10-fold cross-validation for all model. Through SHAP summary plots, model interpretation is achieved at two scales: overall feature importance (global) and individual prediction explanations (local), with feature importance ranked by descending mean SHAP values.

The associations between multiple PFC exposures and asthma, wheeze, FEV_1_, FVC, and FEV₁/FVC were examined using Bayesian Kernel Machine Regression (BKMR) and Weighted Quantile Sum (WQS) regression. BKMR was applied to explore the potential nonlinear and non-additive joint effects of PFC mixtures. BKMR is a Bayesian semiparametric model that flexibly estimates exposure-response relationships and potential interactions among mixture components [27]. In addition, WQS regression was used as a mixture analysis method to construct a weighted index of exposures, ranking them into quantiles and estimating their joint association with the outcomes [28]. WQS assumes directional homogeneity (i.e., all exposures within the index affect the outcome in the same direction) and allows identification of the relative contribution of each chemical to the overall mixture effect. The WQS index was constructed using bootstrap sampling, with 40% of the data allocated for training and 60% for validation. Mediation analysis were analyzed by using R package “mediation”, which implemented causal mediation analysis. Data analysis was carried out in R software (version 4.4.3) and the significance level was set at **P* *< 0.05 for all hypothesis tests.

## 3. Results

### 3.1. The characteristics of population and the distribution of PFC levels in the serum

Table 1 showed the characteristics of the 976 participants aged 12−19 years old from NHANES (2007−2012) included in our study. The median (interquartile range, IQR) age of eligible participants was 15 (14−17) years, with 45.18% females and 54.82% males. Clinically, the median level of total bilirubin (0.7 mg/dL) in this study was within the reference physiological range (≤ 1.34 mg/dL) [29], and the median GGT (14 U/L) was also within the reference physiological range (8−40 U/L for males, 6−26 U/L for females) [30], suggesting no widespread, severe hepatic oxidative stress in the cohort. The detection rates of serum PFCs in our studies were 100% for PFOA, 100% for PFNA, 100% for PFOS, 99.3% for PFHS, 83.8% for PFDE, 76.7% for MPAH, and 54.8% for PFUA, respectively. Among the measured PFAS, PFOS exhibited the highest median level of serum (6.495 ng/mL, IQR: 4.0–10.4), followed by PFOA (2.7 ng/mL, IQR: 1.8–3.7), PFHS (1.5 ng/mL, IQR: 0.8–2.9) and PFNA (0.984 ng/mL, IQR:0.71–1.394). In contrast, PFDE and MPAH showed lower concentrations, with medians of 0.2 ng/mL (IQR: 0.14–0.3) and 0.2 ng/mL (IQR: 0.1–0.3625), respectively. Spearman correlations among serum PFCs were presented in S1 Fig.

**Table 1:** Demographic characteristics of participants aged 12-19 years with available data in the NHANES 2007-2012 cycles (N = 976).

	All	Male	Female
	(N = 976)	(N = 535)	(N = 441)
**Age (years),** **Median (IQR)**	15.0 (14.0, 17.0)	15.0 (14.0, 17.0)	16.0 (13.0, 17.0)
**Race/Ethnicity (%)**			
Mexican American	25.6	26.2	24.9
Other Hispanic	11.7	10.8	12.7
Non-Hispanic White	28.8	30.3	27.0
Non-Hispanic Black	24.7	24.1	25.4
Other Race – Including Multi-Racial	9.2	8.6	10.0
**BMI (kg/m**^**2**^**),** **Median (IQR)**	22.84 (19.8, 27.32)	23.36 (20.28, 27.725)	22.37 (19.49, 26.98)
**Oxidative Stress, Median (IQR)**			
Total Bilirubin, (mg/dL)	0.7 (0.5, 0.9)	0.8 (0.6, 1)	0.6 (0.5, 0.8)
Gamma glutamyl transferase, (U/L)	14 (11, 18)	15 (12, 19.75)	12 (10, 15)
**Inflammatory Markers, Median (IQR)**			
NLR	1.60 (1.17, 2.11)	1.52 (1.13, 2.04)	1.67 (1.23, 2.13)
SII	398.2 (283.2, 555.6)	363.7 (265.9, 517.6)	435.6 (314.7, 615.8)
SIRI	0.80 (0.53, 1.20)	0.79 (0.52, 1.19)	0.85 (0.55, 1.23)
**Serum PFAS level (ng/mL), Median (IQR)**			
PFOA	2.7 (1.8, 3.7)	2.9 (2.905, 4.05)	2.36 (1.6, 3.3)
PFNA	0.984 (0.71, 1.394)	1.066 (0.75, 1.394)	0.902 (0.656, 1.312)
PFDE	0.2 (0.14, 0.3)	0.2 (0.14, 0.3)	0.2 (0.14, 0.3)
PFHS	1.5 (0.8, 2.9)	1.8 (1, 3.485)	1.18 (0.65, 2.3)
PFOS	6.495 (4, 10.4)	7.1 (4.6, 11.3)	5.8 (3.49, 9.36)
MPAH	0.2 (0.1, 0.3625)	0.2 (0.1, 0.4)	0.2 (0.1, 0.3)
**Lung Function, Median (IQR)**			
FVC	3831(3254, 4675.5)	4437(3787.5, 5124.5)	3385 (3025, 3769)
FEV_1_	3307.5(2816.5, 3984)	3842(3166.5, 4400.5)	2966 (2662, 3287)
FEV_1_/FVC	0.87 (0.82, 0.91)	0.86 (0.81, 0.89)	0.88 (0.84, 0.92)
**Wheeze (%)**			
Yes-1	10.7	12.1	8.8
No-2	89.3	87.9	91.2
**Asthma (%)**			
Yes-1	19.6	23.0	15.4
No-2	80.3	76.8	84.6
Don’t know-9	0.1	0.2	0.0

Note: Reference range were ≤ 1.34 mg/dL for total bilirubin and 8–40 U/L (males) or 6–26 U/L (females) for GGT. NLR, Neutrophil to lymphocyte ratio; SII, Systemic immune inflammation index; SIRI, Systemic inflammatory response index.

### 3.2. Associations between serum PFCs and pulmonary function and wheeze and asthma

We analyzed the relationship between serum PFCs and pulmonary function indices and wheeze and asthma (Table 2). In serum PFCs, PFOA was positively correlated with FVC (*β* = 68.85 (40.1,97.59), *P* < 0.001) and FEV_1_ (*β* = 42.37 (17.32,67.41), *P* < 0.001), and negatively correlated with FEV_1_/FVC (*β* = −0.31 (−0.58,-0.04), *P* = 0.025); PFHS was positively correlated with FEV_1_ (*β* = 8.96 (0.31,17.62), *P* = 0.042) and FVC (*β* = 14.4 (4.43, 24.37), *P* = 0.005); PFOS was positively correlated with FEV_1_ (*β* = 6.53 (0.28,12.79), *P* = 0.041) and FVC (*β* = 10.15 (2.94,17.36), *P* = 0.006); MPAH was positively correlated with FVC (*β* = 114.63 (7.83, 221.43), *P* = 0.035) and wheeze (*β* = 0.05 (0, 0.1), *P* = 0.043) after covariate adjustment for confounding.

**Table 2:** Association of serum PFCs with lung health parameters.

PFAS	FEV_1_		FVC		FEV_1_/ FVC		Wheeze		Asthma	
	Adjusted β (95% CI)	*p*	Adjusted β (95% CI)	*p*	Adjusted β (95% CI)	*p*	Adjusted β (95% CI)	*p*	Adjusted β (95% CI)	*p*
PFOA	42.37 (17.32,67.41)	< 0.001***	68.85 (40.1,97.59)	< 0.001***	−0.31 (−0.58, −0.04)	0.025*	0.01 (0,0.02)	0.068	0.01 (−0.01,0.03)	0.302
PFNA	20.81 (−34.37,75.99)	0.459	22.42 (−41.27,86.11)	0.49	0.13 (−0.46,0.72)	0.67	−0.02 (−0.04,0.01)	0.292	0 (−0.03,0.04)	0.805
PFDE	−9.19 (−248.37,229.99)	0.94	61.3 (−214.73,337.33)	0.663	−1.32 (−3.88,1.25)	0.315	0.03 (−0.09,0.15)	0.638	0.09 (−0.07,0.24)	0.273
PFHS	8.96 (0.31,17.62)	0.042*	14.4 (4.43,24.37)	0.005**	−0.05 (−0.14,0.04)	0.305	0 (0,0.01)	0.767	0 (0,0.01)	0.691
PFOS	6.53 (0.28,12.79)	0.041*	10.15 (2.94,17.36)	0.006**	−0.04 (−0.1,0.03)	0.29	0 (0,0)	0.575	0 (−0.01,0)	0.655
MPAH	69.15 (−23.49,161.79)	0.143	114.63 (7.83,221.43)	0.035*	−0.49 (−1.49,0.5)	0.333	0.05 (0,0.1)	0.043*	−0.02 (−0.08,0.04)	0.439

Models were adjusted for age, gender, race and BMI.

And the population was stratified according to gender to evaluate whether the association between serum PFCs and lung health differed between males and females. Age-stratified analyses revealed that PFOA was positively associated with FEV_1_ and FVC in 12–15-year-old adolescents; PFOA was positively associated with wheeze and FVC, and negatively associated with FEV_1_/FVC in 16–19-year-old adolescents. Sex-stratified analyses revealed that PFOA, PFHS, PFOS, MPAH were significantly associated with lung function (FEV₁ and FVC) in females and PFOA was significantly associated with FVC in males (S1 Table).

### 3.3. Restricted cubic spline models examined the dose-response relationship between serum PFC and pulmonary function and wheeze and asthma

**Fig 1:**
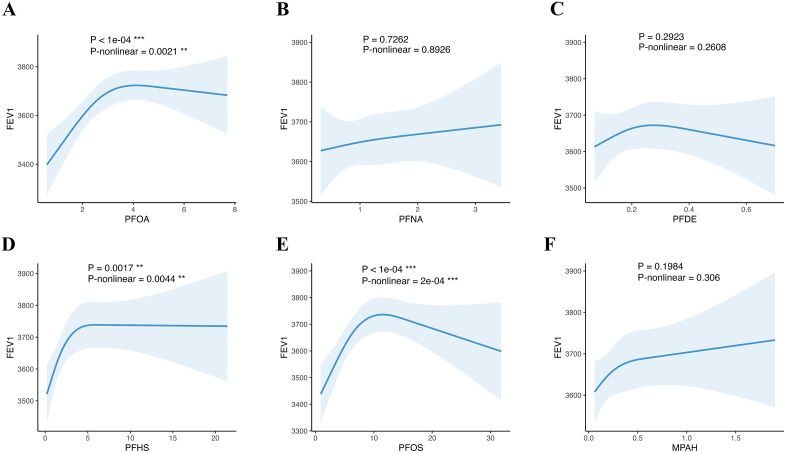
Relationships between serum PFC concentrations and FEV_1_ from restricted cubic splines. Three knots of RCS were located at 10th, 50th, and 90th. The dose-response relationships between PFC and FEV_1_ were: PFOA-FEV_1_, nonlinear; PFHS-FEV_1_, nonlinear; PFOS-FEV_1_, nonlinear.

**Fig 2:**
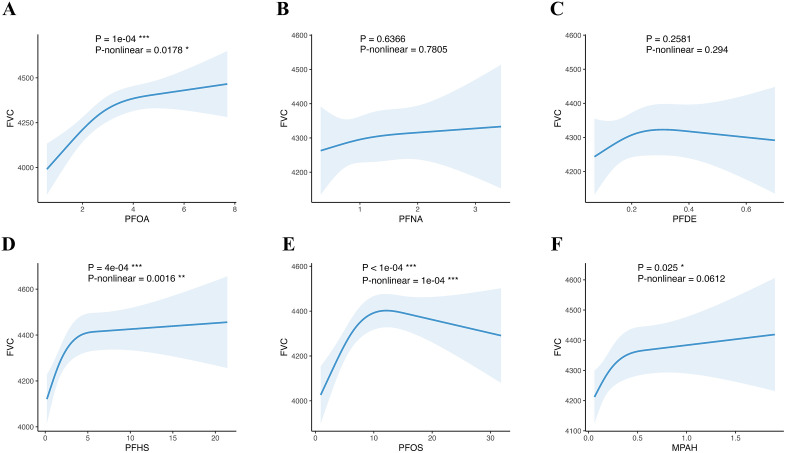
Relationships between serum PFC concentrations and FVC from restricted cubic splines. Three knots of RCS were located at 10th, 50th, and 90th. The dose-response relationships between PFC and FVC were: PFOA-FVC, nonlinear; PFHS-FVC, nonlinear; PFOS-FVC, nonlinear; MPAH-FVC, linear.

Restricted cubic spline models were utilized to examine the dose-response relationships between serum PFC concentrations and pulmonary function (FEV₁, FVC, FEV₁/FVC ratio) as well as respiratory outcomes (wheeze and asthma). As shown in Figs 1 and 2, PFOA, PFHS and PFOS exhibited significant nonlinear dose-response relationships with FEV₁ and FVC. PFOA and PFOS showed an inverse U-shaped association with asthma. And PFHS showed a positive dose-response relationship with FEV₁ and FVC. MPAH demonstrated a positive linear association with FVC. And MPAH demonstrated a linear association with FVC. However, no significant dose-response relationships were observed for FEV₁/FVC ratio, wheeze, or asthma with any serum PFCs (S2–S4 Figs).

### 3.4. The joint effectiveness of serum PFC for respiratory health analyzed by Bayesian kernel machine regression and weighted quantile sum regression

**Fig 3:**
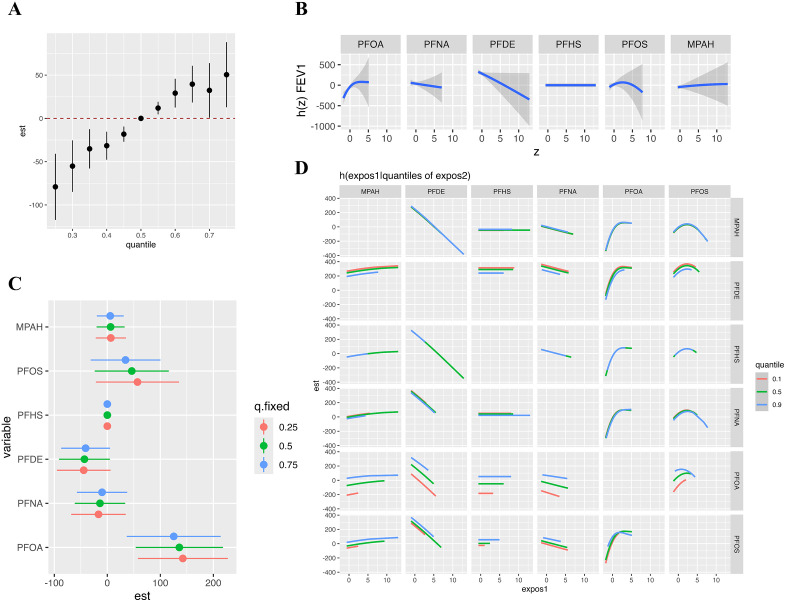
The effects of serum PFCs on FEV_1_ was estimated by BKMR models. **(A)** The joint effect of mixed exposure on FEV_1_ compared with that at certain percentiles with those at the 50th percentile; **(B)** The effect of individual PFC exposures on FEV_1_ along with their respective 95% confidence intervals; **(C)** The univariate risk for individual PFC exposures by quartiles; **(D)** Bivariate exposure-response at the 10% (red line), 50% (green line), and 90% (blue line) percentiles with all other PFCs set at their respective medians. Model was adjusted for age, gender, race and BMI.

**Fig 4:**
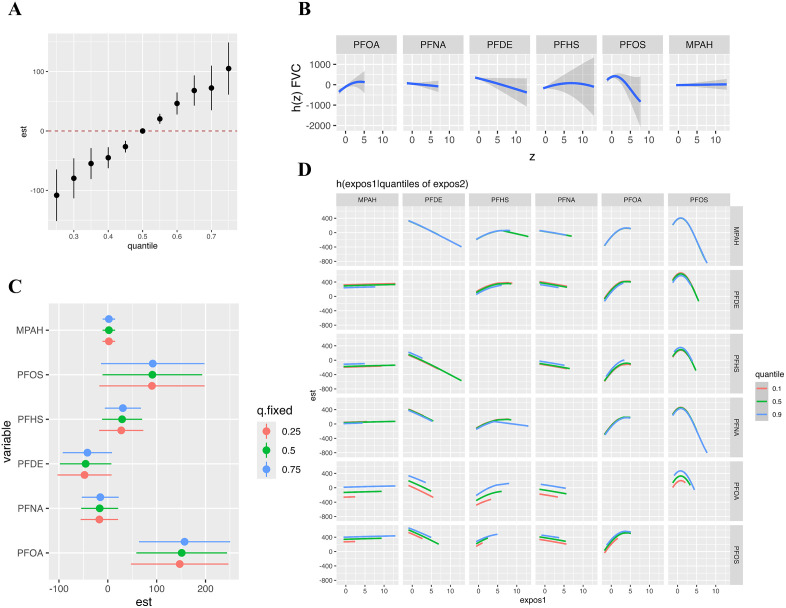
The effects of serum PFCs on FVC was estimated by BKMR models. **(A)** The joint effect of mixed exposure on FVC compared with that at certain percentiles with those at the 50th percentile; **(B)** The effect of individual PFC exposures on FVC along with their respective 95% confidence intervals; **(C)** The univariate risk for individual PFC by quartiles; **(D)** Bivariate exposure-response at the 10% (red line), 50% (green line), and 90% (blue line) percentiles with all other PFCs set at their respective medians. Model was adjusted for age, gender, race and BMI.

**Fig 5:**
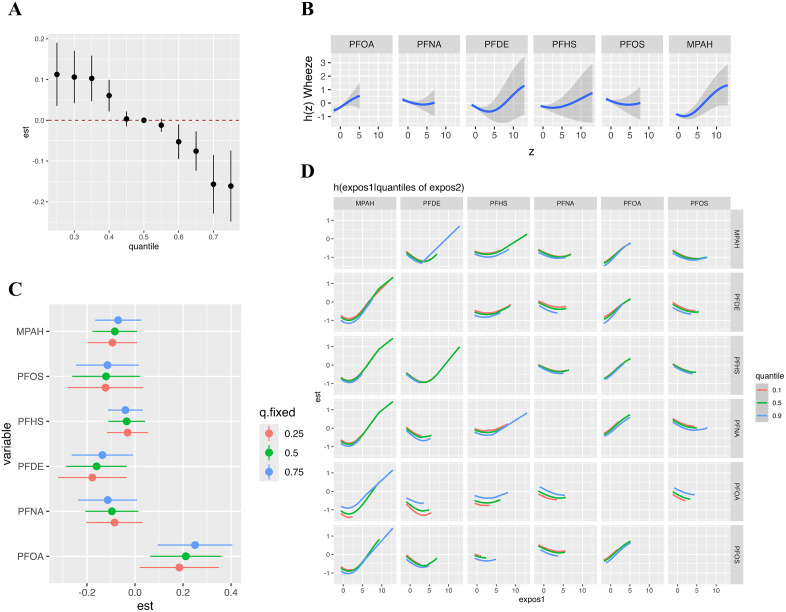
The effects of serum PFCs on wheeze risk was estimated by BKMR models. **(A)** The joint effect of mixed exposure on wheeze risk compared with that at certain percentiles with those at the 50th percentile; **(B)** The effect of individual PFC exposures on wheeze risk along with their respective 95% confidence intervals; **(C)** The univariate risk for individual PFC by quartiles; **(D)** Bivariate exposure-response at the 10% (red line), 50% (green line), and 90% (blue line) percentiles with all other PFCs set at their respective medians. Model was adjusted for age, gender, race and BMI.

We employed Bayesian kernel machine regression and weighted quantile sum regression to assess the joint effects of serum PFC mixtures on pulmonary function (FEV₁, FVC, FEV₁/FVC) and respiratory outcomes (wheeze, asthma). PFC co-exposure was significantly correlated with increased FEV₁ (Fig 3) and FVC (Fig 4). Conversely, the mixture showed a negative association with wheeze prevalence (Fig 5), indicating reduced odds of wheeze at higher levels of PFC. No significant linkages were identified between PFC mixtures and FEV₁/FVC and asthma risk in either model (S5 and S6 Figs). The weights of individual PFCs in the WQS model, indicating their contribution to the overall mixture effect, were shown in S7 Fig.

### 3.5. Variable selection

**Fig 6:**
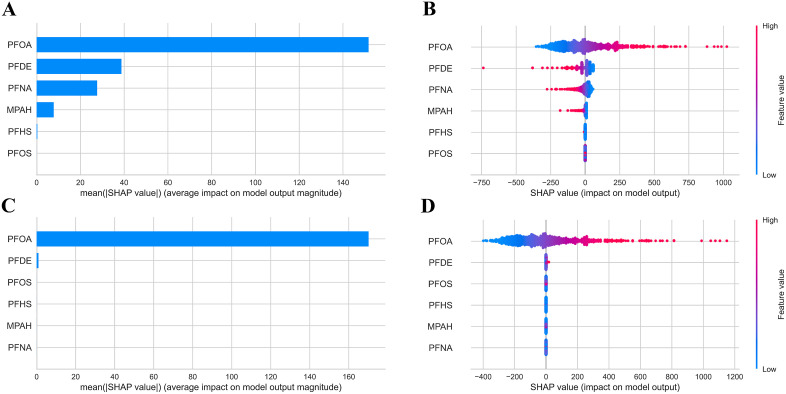
Variables importance rankings measuring the association between PFCs exposure and lung functions, wheeze and asthma based on ML models interpreted with the SHAP approach. **(A)** Bar plot for FEV_1_ based on Lasso Regression model; **(B)** Dot plot for FEV_1_ based on Lasso Regression model; **(C)** Bar plot for FVC based on Automatic Relevance Determination model; **(D)** Dot plot for FVC based on Automatic Relevance Determination model.

S2–S6 Tables showed the performance of the ML models output by the “pycaret”. Combined R^2^ or AUC, Lasso regression had the best regression performance for FEV_1_, automatic relevance determination for FVC, support vector regression for FEV_1_/FVC, and logistic regression for wheeze and asthma, respectively. To interpret the final model, the SHAP method was applied to assess the predictive contribution of each serum PFC. For example, the model suggested that individuals with higher PFOA levels were more likely to exhibit greater FEV₁ and FVC values while PFDE has the opposite effect (Fig 6). Although logistic regression had the best performance in current methods for wheeze and asthma, the AUC was less than 0.6 (S9 Fig). It is important to note that given this poor performance, these models are for exploratory analysis and aims to identify potential exposure-outcome relationships within a complex mixture.

### 3.6. Identification of potential mediators using mediation analysis

S7 Table presented the relationship among serum PFC and immune parameters as well as oxidative stress indicator. PFOA was associated with total bilirubin. Meanwhile, NLR, SIRI, gamma-glutamyl transferase and total bilirubin level were correlated with FVC and FEV_1_ (S8 and S9 Tables). The outcomes of the mediation analysis indicated that total bilirubin explained 8.2% of the effect of PFOA on FEV_1_ (indirect effect *β* = 3.55, 95% CI: 0.51, 7.35; total effect *β* = 42.10). Similarly, it mediated 6.8% of the effect of PFOA on FVC (indirect effect *β* = 4.73, 95% CI: 0.84, 10.10; total effect *β* = 69.05) and 9.0% of the effect of PFOS on FVC (indirect effect *β* = 0.94, 95% CI: 0.01, 2.13; total effect *β* = 10.15).

### 3.7. Screening for candidate target genes associated with PFC exposure, pulmonary function impairment and asthma

**Fig 7:**
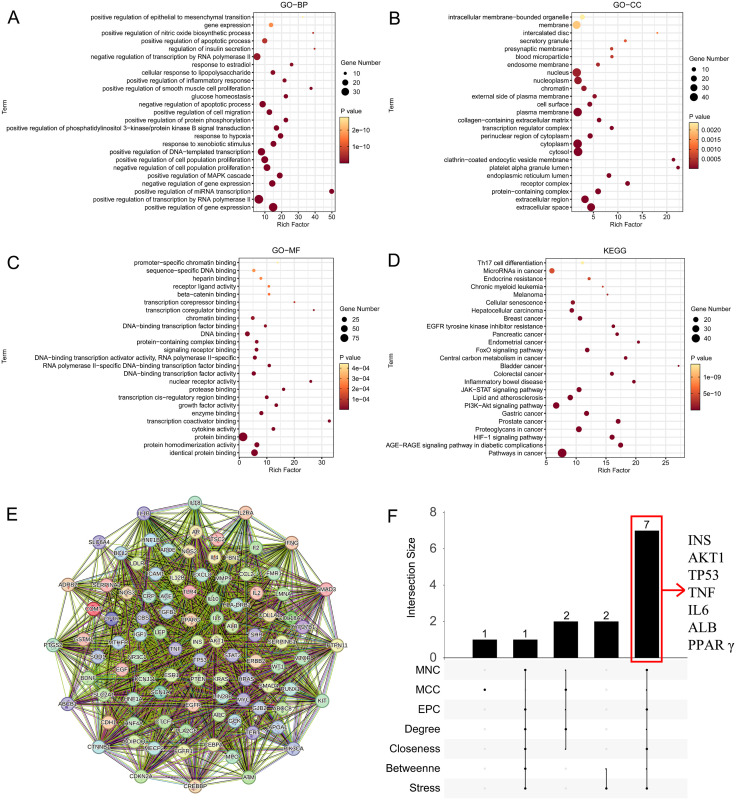
Exploration of the molecular mechanism by which serum PFCs metabolites influence lung health. **(A)** Bubble diagram of BP in GO enrichment analysis; **(B)** Bubble diagram of CC in GO enrichment analysis; **(C)** Bubble diagram of MF in GO enrichment analysis; **(D)** Bar plot of KEGG enrichment analysis; **(E)** The PPI network of overlapping targets; **(F)** UpSet plot of 7 topological algorithms.

**Fig 8:**
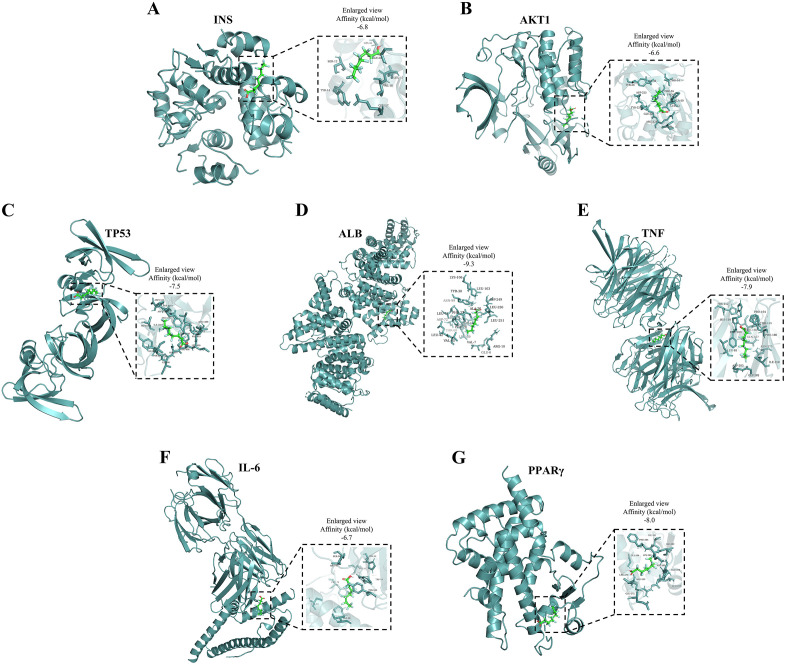
Molecular docking in each protein with the PFCs. **(A)** Molecular docking of PFOA and INS protein; **(B)** Molecular docking of PFOA and AKT1 protein; **(C)** Molecular docking of PFOA and TP53 protein; **(D)** Molecular docking of PFOA and ALB protein; **(E)** Molecular docking of PFOA and TNF protein; **(F)** Molecular docking of PFOA and IL-6 protein; **(G)** Molecular docking of PFOA and PPARγ protein.

Total of 12593 PFCs-related genes were filtered from the comparative toxicogenomics database and 133 genes were highly related with inflammation as well as 924 genes were highly associated with lung function impairment in teenagers through the GeneCard database (relevance scores>50). Finally, we screened 97 cross-targets as targets for PAHs-induced pulmonary function impairment in teenagers. Gene ontology and Kyoto encyclopedia of genes and genomes analysis of the 25 targets based on the DAVID databases were performed (Fig 7A–7D). The results of pathway enrichment indicated that these genes were related with pathways in cancer, and Th17 cell differentiation. We constructed the protein-protein interaction network to show the interactions of targets by Cytoscape (Fig 7E). Then the 7 topological information was used to further screen the target genes and seven hub genes (INS, AKT1, TP53, TNF, IL6, ALB and PPARγ) were marked (Fig 7F). We also investigated the interaction between PFOA and seven hub proteins using molecular docking analysis (Fig 8). The binding affinity of PFOA with PPARγ and ALB was estimated to range from −7.5 kcal/mol to −9.0 kcal/mol, and from −8 kcal/mol to −10 kcal/mol, respectively, indicating moderate to strong spontaneous binding.

## 4. Discussion

Our analysis of NHANES data (2007–2012) revealed that serum PFCs, notably PFOA, PFOS, PFHS, and MPAH, were positively associated with lung function parameters (FEV₁ and FVC) in adolescents, particularly in females, and PFOA and MPAH exposure was significantly associated with an increased risk of wheeze specifically in older adolescents aged 16–19 years. Intriguingly, higher PFC concentrations exhibited a nonlinear dose-response relationship with improved FEV₁ and FVC. Conversely, PFOA was negatively associated with FEV₁/FVC, suggesting potential airway obstruction effects. Mediation analysis implicated total bilirubin in partially explaining the PFC-lung function relationship, and gene enrichment studies identified pathways related to inflammation (e.g., Th17 differentiation) and key hub genes (e.g., IL6, TNF, PPARγ) potentially linking PFC exposure to lung health.

These results were inconsistent with a meta-analysis which reported PFC-associated declines in childhood lung function and increased asthma risk [31]. However, our findings aligned with emerging evidence suggesting that PFC effects may vary by age, exposure window, and sex. For instance, the sex-specific associations-stronger in females was observed, a finding aligned with sex-stratified immune responses to PFCs in adolescents reported by Pan et al. [32]. Meanwhile, the stronger associations in females also were found in the COPSAC2010 cohort, where prenatal PFC exposure was linked to altered immune profiles in girls but not boys [33]. Hormonal interactions, particularly estrogen’s role in modulating PPARγ and oxidative stress pathways, may explain this sex disparity. For instance, estrogen enhances PPARγ expression, potentially amplifying anti-inflammatory responses to PFAS in females [32]. Conversely, PFCs are known endocrine disruptors, and their interference with hormone signaling could disproportionately affect females during puberty-a critical window for lung development [34]. Such discrepancies emphasized the need to consider demographic and developmental factors in PFC toxicity assessments.

The lack of significant associations between serum PFCs and asthma aligned with several studies [35,36] but contrasted with others [37,38]. This inconsistency may stem from heterogeneity in asthma phenotypes, exposure timing (prenatal vs. postnatal), or outcome measurement (e.g., self-reported wheeze vs. clinician-diagnosed asthma). Our reliance on NHANES data, which uses self-reported wheeze and asthma, may underestimate true associations due to misclassification bias. Furthermore, the low AUC (<0.6) for asthma models highlighted the limitations of current PFC biomarkers in predicting complex multifactorial diseases. We further explored the association between serum PFC levels and health indicators in 12–19 aged children and adolescents(n = 3352) using data from the 2013–2014, 2015–2016, and 2017–2018 NHANES circles. However, the data from these circles did not included three key spirometric parameters. S9 Table showed demographic characteristics of participants aged 12–19 years with available data in the NHANES 2013–2018 cycles. Age-stratified analyses revealed that PFDE (OR=1.22, *P* = 0.036) and MPAH (OR=1.152, *P* = 0.038) was positively associated with asthma in 12–15-year-old adolescents. Sex-stratified analyses revealed that PFDE were significantly associated with asthma in males (OR=1.19, *P* = 0.046). PFOA exhibited significant nonlinear dose-response relationships with asthma in 12–15-year-old adolescents in restricted cubic spline models (S9 Fig). There were no joint effects of serum PFC mixtures on asthma by Bayesian kernel machine regression and weighted quantile sum regression. And also, there were no mediating effects of immune parameters as well as oxidative stress indicator between serum PFC and asthma. However, studies using murine asthma models demonstrate that PFC exposure induces asthma-related outcomes, including airway hyperresponsiveness and enhanced inflammatory responses [11]. Early-life exposure to PFCs in mice attenuated airway antigen bioactivity, impairing the modulation of pulmonary inflammatory responses and posing risks to lung development. Specifically, PFOS exhibited high-affinity binding to the major HDM allergen Der p1 and the lipid A moiety of lipopolysaccharide, resulting in the functional inactivation of both antigens [39]. These findings suggested that the potential impact of serum PFC exposure on asthma pathogenesis warrants further investigation through a comprehensive approach combining robust epidemiological studies with carefully designed animal experiments.

Our gene enrichment and molecular docking analyses provide a tentative roadmap for mechanistic research. The identification of Th17 differentiation and cancer-related pathways aligns with experimental evidence linking serum PFC to Th17/regulatory T-cell imbalance—a hallmark of allergic inflammation [40]. Similarly, hub genes like IL6 and TNF, central to inflammatory cascades, were prioritized in protein-protein interaction networks. Molecular docking results further suggested that serum PFC may directly interact with proteins such as PPARγ and ALB, potentially disrupting lipid metabolism and inflammatory signaling. These findings resonate with Solan and Park’s [41] call for leveraging route-to-route extrapolation for risk assessment to unravel PFAS toxicity mechanisms.

There was some limitation in this study. (1) The study used cross-sectional data from NHANES (2007–2012), which limits our ability to establish temporal relationships or infer causality. Longitudinal studies are needed to confirm the directionality of these associations. (2) Although we adjusted for key demographic covariates (e.g., age, sex, BMI), unmeasured confounders (e.g., diet, indoor air pollution, genetic susceptibility) may still influence the observed associations. (3) PFC exposure was assessed using single-timepoint serum measurements, which may not accurately reflect long-term exposure patterns, especially for short-chain PFC (e.g., PFBA) with shorter biological half-lives. Intra-individual variability in PFC levels over time could lead to exposure misclassification, potentially biasing results toward the null. Future studies should incorporate repeated exposure measurements to improve accuracy. (4) It must be acknowledged that the network toxicology analyses in this study serve as a supportive component rather than a core mechanistic investigation. While these analyses provide valuable insights into the potential toxicity pathways and molecular mechanisms underlying PFC-induced lung function impairment, they do not constitute novel mechanistic evidence and should be interpreted as hypothesis-generating for future research.

## 5. Conclusion

Our results demonstrated that serum PFC exposure contributes to impaired respiratory health among vulnerable groups, suggesting that PFC effects are dose-, age-, sex-, and outcome-specific. These findings underscore the importance of addressing environmental exposures during early life stages to mitigate the burden of adult respiratory diseases.

## Supporting information

S1 Fig Spearman correlation coefficients between different serum PFCs. (TIF)

S2 Fig Relationships between serum PFC concentrations and FEV_1_/FVC from restricted cubic splines. Three knots of RCS were located at 10th, 50th, and 90th.
(TIF)

S3 Fig Relationships between serum PFC concentrations and wheeze risk from restricted cubic splines. Three knots of RCS were located at 10th, 50th, and 90th.
(TIF)

S4 Fig Relationships between serum PFC concentrations and asthma risk from restricted cubic splines. Three knots of RCS were located at 10th, 50th, and 90th.
(TIF)

S5 Fig The effects of serum PFCs on FEV_1_/FVC was estimated by BKMR models. (A) The joint effect of mixed exposure on FEV_1_/FVC compared with that at certain percentiles with those at the 50th percentile; (B) The effect of individual PFC exposures on FEV_1_/FVC along with their respective 95% confidence intervals; (C) The univariate risk for individual PFC by quartiles; (D) Bivariate exposure-response at the 10% (red line), 50% (green line), and 90% (blue line) percentiles with all other PFCs set at their respective medians. Model was adjusted for age, gender, race and BMI.
(TIF)

S6 Fig The effects of serum PFCs on asthma risk was estimated by BKMR models. (A) The joint effect of mixed exposure on asthma risk compared with that at certain percentiles with those at the 50th percentile; (B) The effect of individual PFC exposures on asthma risk along with their respective 95% confidence intervals; (C) The univariate risk for individual PFC by quartiles; (D) Bivariate exposure-response at the 10% (red line), 50% (green line), and 90% (blue line) percentiles, with all other PFCs set at their respective medians. Model was adjusted for age, gender, race and BMI.
(TIF)

S7 Fig Association between serum PFC levels and FEV_1_ (A), FVC (B), FEV_1_/FVC (C), wheeze (D) and asthma (E) based on weighted quantile sum (WQS) regression analysis. (TIF)

S8 Fig Variables importance rankings for the association between PFCs exposure and lung functions, wheeze and asthma based on ML models interpreted with the SHAP approach. (A) Bar plot for FEV_1_/FVC based on Support Vector Regression model; (B) Dot plot for FEV_1_/FVC based on Support Vector Regression model; (C) Bar plot for wheeze based on Logistic Regression model; (D) Dot plot for wheeze based on Logistic Regression model; (E) Bar plot for asthma based on Logistic Regression model; (F) Dot plot for asthma based on Logistic Regression model.
(TIF)

S9 Fig Relationships between PFOA concentrations and asthma risk from restricted cubic splines. Three knots of RCS were located at 10th, 50th, and 90th.
(TIF)

S1 Table Associations of serum PFCs with lung health stratified by age and gender in the NHANES 2007–2012. (DOCX)

S2 Table Performance of the machine learning model for regression of “FEV_1_”. (DOCX)

S3 Table Performance of the machine learning model for regression of “FVC”. (DOCX)

S4 Table Performance of the machine learning model for regression of “FEV_1_/FVC”. (DOCX)

S5 Table Performance of the machine learning model for classification of “Wheeze”. (DOCX)

S6 Table Performance of the machine learning model for classification of “Asthma”. (DOCX)

S7 Table Association between serum PFCs and immune indices and oxidative stress index. (DOCX)

S8 Table Association between immune indices and oxidative stress index and lung health parameters. (DOCX)

S9 Table Demographic characteristics of participants aged 12‐19 years with available data in the NHANES 2013‐2018 cycles (N = 3352). (DOCX)

## Abbreviations

PFCs: Perﬂuorinated chemicals
FEV_1_: forced expiratory volume in 1 second
FVC: forced vital capacity
PFOA: perfluorooctanoic acid
PFHS: perfluorohexane sulfonic acid
PFOS: perfluorooctane sulfonic acid
MPAH: 2-(N-Methyl-perfluorooctane sulfonamido) acetic acid
PFNA: perfluorononanoic acid
INS: insulin
AKT1: v-akt murine thymoma viral oncogene homologue 1
TP53: tumor protein p53
TNF: tumor necrosis factor
IL6: interleukin-6
ALB: albumin
PPARγ: peroxisome proliferator-activated receptor gamma
NHANES: nutrition examination survey
SPE-HPLC-TIS-MS/MS: solid phase extraction coupled to High Performance Liquid Chromatography-Turbo Ion Spray ionization-tandem Mass Spectrometry
PFHP: perfluoroheptanoic acid
PFDE: perfluorodecanoic acid
PFUA: perfluoroundecanoic acid
PFDO: perfluorododecanoic acid
PFBS: perfluorobutane sulfonic acid
PFSA: perfluorooctane sulfonamide
EPAH: 2-(N-ethyl-perfluorooctane sulfonamido) acetic acid
LOD: the limit of detection
COPD: chronic obstructive pulmonary disease
NLR: Neutrophil-to-lymphocyte ratio
SII: immune-inflammation index
SIRI: systemic inflammation response index
GO: Gene Ontology
KEGG: Kyoto Encyclopedia of Genes and Genomes
PPI: protein-protein interaction network
MCC: maximal clique centrality
MNC: maximum neighborhood component
EPC: edge percolated component
RCSB: research collaboratory for structural bioinformatics database
SD: standard deviation
OR: odds ratio
CI: confidence interval
RCS: restricted cubic spline
ML: machine learning
SHAP: SHapley Additive explanation
BKMR: Bayesian Kernel Machine Regression
WQS: Weighted Quantile Sum
IQR: interquartile range
AUC: area under the curve
HDM: House Dust Mite
Der p1: Dermatophagoides pteronyssinus group 1 antigen
LPS: lipopolysaccharide
BMI: body mass index.
