# Supplementary material for: Perfluorinated chemicals and adolescent respiratory health: Epidemiological evidence and mechanistic insights
Source: PLoS One. 2025 Nov 14;20(11):e0336788. doi: 10.1371/journal.pone.0336788 (PMC12617853; doi:10.1371/journal.pone.0336788)
Supplement: S1 Table — (DOCX) [file pone.0336788.s010.docx]

**Perfluorinated chemicals and adolescent respiratory health: Epidemiological evidence and mechanistic insights**

Xinfeng Xu^¶^, Xinyao Jiang^¶^, Meng Zou, Jinyan Hui, Guang Huang^*^, [Qian Wu](https://pubmed.ncbi.nlm.nih.gov/?term=Wu+Q&cauthor_id=36136199)^*^

China International Cooperation Center (CCC) for Environment and Human Health and Department of Health Inspection and Quarantine, School of Public Health, Nanjing Medical University, Nanjing, China.

E-mail addresses: scottsmith@stu.njmu.edu.cn (X. Xu), jiang_xy0604@stu.njmu.edu.cn (X. Jiang), 2022121213@stu.njmu.edu.cn (M. Zou), 2024120805@stu.njmu.edu.cn (J. Hui), guanghuang@njmu.edu.cn (G. Huang), wuqian@njmu.edu.cn (Q. Wu).

^*^Corresponding authors: wuqian@njmu.edu.cn (Q. Wu); guanghuang@njmu.edu.cn (G. Huang).

^¶^Co-first authors have equal contributions to the work.

**Highlights**

- **The serum PFCs were associated with lung health among adolescents.**
- **PFOA was the dominant contributor in mixed PFC exposures.**
- **Oxidative stress may be contributed to PFC-related respiratory toxicity.**

**S1 Table. Associations of serum PFCs with lung health stratified by age and gender in the NHANES 2007-2012**

|  | **FEV_1_** | | **FVC** | | **FEV_1_/FVC** | | **Wheeze** | | **Asthma** | |
| --- | --- | --- | --- | --- | --- | --- | --- | --- | --- | --- |
|  | Adjusted β  (95% CI) | *p* | Adjusted β  (95% CI) | *p* | Adjusted β  (95% CI) | *p* | Adjusted β  (95% CI) | *p* | Adjusted β  (95% CI) | *p* |
| **PFOA** | | | | | | | | | | |
| **Age 12-15** | 41.15 (0.8,81.5) | 0.046* | 50.54 (5.42,95.66) | 0.028* | -0.08 (-0.47,0.31) | 0.694 | 0 (-0.01,0.02) | 0.62 | 0 (-0.02,0.03) | 0.89 |
| **Age 16-19** | 51.59 (-1.56,104.74) | 0.057 | 101.03 (35.29,166.77) | 0.003** | -0.75 (-1.19,-0.3) | 0.001** | 0.03 (0.01,0.05) | 0.01* | 0.01 (-0.01,0.04) | 0.279 |
| **Male** | 25.47 (-8.91,59.84) | 0.146 | 56 (16.72,95.27) | 0.005** | -0.36 (-0.73,0) | 0.049* | 0.01 (0,0.03) | 0.146 | 0 (-0.02,0.03) | 0.732 |
| **Female** | 60.02 (28.8,91.23) | <0.001*** | 77.36 (40.99,113.73) | <0.001*** | -0.23 (-0.63,0.18) | 0.273 | 0.01 (-0.01,0.03) | 0.258 | 0.02 (-0.01,0.04) | 0.177 |
| **PFNA** | | | | | | | | | | |
| **Age 12-15** | 0.81 (-80.88,82.5) | 0.984 | -18.27 (-109.67,73.13) | 0.695 | 0.36 (-0.43,1.16) | 0.369 | -0.03 (-0.07,0.01) | 0.186 | 0.02 (-0.03,0.07) | 0.49 |
| **Age 16-19** | 162.39 (-259.35,584.13) | 0.45 | 79.33 (-421.76,580.42) | 0.756 | 2.37 (-1.21,5.95) | 0.195 | -0.02 (-0.19,0.15) | 0.816 | 0.08 (-0.12,0.28) | 0.447 |
| **Male** | 15.69 (-58.42,89.8) | 0.678 | 15.67 (-69.46,100.81) | 0.718 | 0.19 (-0.59,0.98) | 0.627 | -0.03 (-0.07,0) | 0.077 | -0.02 (-0.07,0.03) | 0.464 |
| **Female** | 27.91 (-43.76,99.59) | 0.444 | 31.48 (-52.34,115.29) | 0.461 | 0.01 (-0.91,0.93) | 0.978 | 0.02 (-0.03,0.06) | 0.425 | 0.05 (-0.01,0.1) | 0.1 |
| **PFDE** | | | | | | | | | | |
| **Age 12-15** | -89.23 (-575.19,396.72) | 0.718 | -131.03 (-674.8,412.75) | 0.636 | 0.22 (-4.5,4.94) | 0.926 | -0.04 (-0.27,0.19) | 0.743 | 0.11 (-0.19,0.41) | 0.464 |
| **Age 16-19** | -10.41 (-418.98,398.16) | 0.96 | 6.09 (-496.46,508.65) | 0.981 | -0.28 (-3.71,3.14) | 0.872 | 0.1 (-0.05,0.24) | 0.201 | 0.09 (-0.11,0.28) | 0.396 |
| **Male** | 148.45 (-255.99,552.89) | 0.471 | 305.55 (-158.53,769.64) | 0.196 | -1.59 (-5.86,2.68) | 0.465 | -0.09 (-0.29,0.11) | 0.386 | 0.03 (-0.23,0.29) | 0.835 |
| **Female** | 22.87 (-217.39,263.12) | 0.852 | 46.2 (-234.73,327.12) | 0.747 | -0.79 (-3.88,2.29) | 0.613 | 0.1 (-0.05,0.25) | 0.194 | 0.13 (-0.05,0.32) | 0.165 |
| **PFHS** | | | | | | | | | | |
| **Age 12-15** | 7.75 (-3.17,18.66) | 0.164 | 9.33 (-2.88,21.55) | 0.134 | 0.01 (-0.09,0.12) | 0.804 | 0 (-0.01,0.01) | 0.943 | 0 (-0.01,0.01) | 0.932 |
| **Age 16-19** | -0.02 (-13.34,13.29) | 0.997 | 4.15 (-12,20.3) | 0.614 | -0.05 (-0.15,0.06) | 0.393 | 0 (0,0.01) | 0.361 | 0 (0,0.01) | 0.518 |
| **Male** | 3.79 (-7.89,15.47) | 0.524 | 10.64 (-2.76,24.03) | 0.119 | -0.08 (-0.2,0.05) | 0.221 | 0 (0,0.01) | 0.414 | 0 (-0.01,0.01) | 0.882 |
| **Female** | 15.57 (4.46,26.68) | 0.006** | 18.33 (5.35,31.32) | 0.006** | -0.01 (-0.15,0.14) | 0.939 | 0 (-0.01,0) | 0.507 | 0 (-0.01,0.01) | 0.619 |
| **PFOS** | | | | | | | | | | |
| **Age 12-15** | 12.33 (2.8,21.85) | 0.011* | 14.12 (3.46,24.78) | 0.01* | 0.01 (-0.09,0.1) | 0.887 | 0 (0,0.01) | 0.669 | 0 (-0.01,0) | 0.487 |
| **Age 16-19** | 78.16 (31.87,124.44) | <0.001*** | 86.69 (31.63,141.74) | 0.002** | 0.07 (-0.33,0.47) | 0.726 | -0.01 (-0.03,0.01) | 0.252 | 0 (-0.02,0.03) | 0.785 |
| **Male** | 1.73 (-6.56,10.02) | 0.681 | 5.2 (-4.32,14.71) | 0.284 | -0.03 (-0.12,0.06) | 0.486 | 0 (0,0) | 0.827 | 0 (-0.01,0) | 0.604 |
| **Female** | 12.18 (3.84,20.52) | 0.004** | 16.07 (6.34,25.79) | 0.001** | -0.06 (-0.17,0.05) | 0.27 | 0 (-0.01,0) | 0.224 | 0 (-0.01,0.01) | 0.978 |
| **MPAH** | | | | | | | | | | |
| **Age 12-15** | 26.47 (-107.08,160.02) | 0.697 | 58.87 (-90.52,208.26) | 0.439 | -0.47 (-1.76,0.83) | 0.48 | 0.06 (0,0.13) | 0.058 | -0.03 (-0.11,0.05) | 0.426 |
| **Age 16-19** | 28 (-116.55,172.55) | 0.704 | 30.02 (-149.69,209.73) | 0.743 | 0.14 (-1.07,1.36) | 0.817 | 0.06 (0.01,0.12) | 0.024* | -0.04 (-0.11,0.03) | 0.303 |
| **Male** | 70.36 (-52.43,193.15) | 0.261 | 128.97 (-11.82,269.77) | 0.073 | -0.55 (-1.85,0.74) | 0.403 | 0.09 (0.03,0.16) | 0.003** | -0.01 (-0.09,0.07) | 0.715 |
| **Female** | 116.96 (-6.42,240.35) | 0.063 | 148.31 (4.12,292.49) | 0.044* | -0.33 (-1.92,1.26) | 0.684 | -0.04 (-0.12,0.03) | 0.256 | -0.04 (-0.14,0.05) | 0.39 |
