# Supplementary material for: Perfluorinated chemicals and adolescent respiratory health: Epidemiological evidence and mechanistic insights
Source: PLoS One. 2025 Nov 14;20(11):e0336788. doi: 10.1371/journal.pone.0336788 (PMC12617853; doi:10.1371/journal.pone.0336788)
Supplement: S2 Table — (DOCX) [file pone.0336788.s011.docx]

**Perfluorinated chemicals and adolescent respiratory health: Epidemiological evidence and mechanistic insights**

Xinfeng Xu^¶^, Xinyao Jiang^¶^, Meng Zou, Jinyan Hui, Guang Huang^*^, [Qian Wu](https://pubmed.ncbi.nlm.nih.gov/?term=Wu+Q&cauthor_id=36136199)^*^

China International Cooperation Center (CCC) for Environment and Human Health and Department of Health Inspection and Quarantine, School of Public Health, Nanjing Medical University, Nanjing, China.

E-mail addresses: scottsmith@stu.njmu.edu.cn (X. Xu), jiang_xy0604@stu.njmu.edu.cn (X. Jiang), 2022121213@stu.njmu.edu.cn (M. Zou), 2024120805@stu.njmu.edu.cn (J. Hui), guanghuang@njmu.edu.cn (G. Huang), wuqian@njmu.edu.cn (Q. Wu).

^*^Corresponding authors: wuqian@njmu.edu.cn (Q. Wu); guanghuang@njmu.edu.cn (G. Huang).

^¶^Co-first authors have equal contributions to the work.

**Highlights**

- **The serum PFCs were associated with lung health among adolescents.**
- **PFOA was the dominant contributor in mixed PFC exposures.**
- **Oxidative stress may be contributed to PFC-related respiratory toxicity.**

**S2 Table. Performance of the machine learning model for regression of “FEV_1_”**

| Abbr. | Model | MAE | MSE | RMSE | R^2^ | RMSLE |
| --- | --- | --- | --- | --- | --- | --- |
| lasso | Lasso Regression | 650.2392 | 638291.498 | 797.3487 | 0.0085 | 0.2373 |
| ridge | Ridge Regression | 650.1326 | 638295.546 | 797.351 | 0.0085 | 0.2373 |
| llar | Lasso Least Angle Regression | 650.2392 | 638291.279 | 797.3486 | 0.0085 | 0.2373 |
| en | Elastic Net | 654.3425 | 639211.896 | 797.7302 | 0.0084 | 0.2375 |
| br | Bayesian Ridge | 654.2947 | 639435.092 | 797.8659 | 0.0081 | 0.2375 |
| lr | Linear Regression | 650.2439 | 638558.106 | 797.5231 | 0.008 | 0.2374 |
| ard | Automatic Relevance Determination | 654.7378 | 640159.238 | 798.5953 | 0.0054 | 0.2376 |
| lar | Least Angle Regression | 650.3978 | 641158.835 | 799.1531 | 0.0038 | 0.2379 |
| huber | Huber Regressor | 645.9954 | 643203.997 | 799.9634 | 0.0025 | 0.2367 |
| omp | Orthogonal Matching Pursuit | 663.8305 | 650522.483 | 804.8497 | -0.0093 | 0.2394 |
| dummy | Dummy Regressor | 668.0312 | 658395.783 | 809.4139 | -0.0197 | 0.2411 |
| gbr | Gradient Boosting Regressor | 654.4629 | 659561.325 | 809.5497 | -0.0239 | 0.2404 |
| ada | AdaBoost Regressor | 666.3704 | 659834.408 | 810.7425 | -0.0253 | 0.2437 |
| svm | Support Vector Regression | 659.7496 | 666878.302 | 813.8063 | -0.03 | 0.2385 |
| rf | Random Forest Regressor | 664.0736 | 681365.119 | 823.4183 | -0.0599 | 0.2443 |
| et | Extra Trees Regressor | 666.97 | 683058.232 | 824.513 | -0.0628 | 0.2443 |
| catboost | CatBoost Regressor | 680.0124 | 717266.813 | 844.4519 | -0.1145 | 0.2506 |
| tr | TheilSen Regressor | 672.4505 | 729644.88 | 852.406 | -0.1359 | 0.2479 |
| knn | K Neighbors Regressor | 687.232 | 743764.684 | 861.0005 | -0.1612 | 0.2543 |
| par | Passive Aggressive Regressor | 693.5464 | 780595.683 | 877.1733 | -0.1973 | 0.2562 |
| lightgbm | Light Gradient Boosting Machine | 693.3262 | 773541.454 | 877.2723 | -0.2019 | 0.2603 |
| xgboost | Extreme Gradient Boosting | 726.9746 | 836701.176 | 912.6576 | -0.3038 | 0.2689 |
| ransac | Random Sample Consensus | 848.2601 | 1343956.56 | 1130.1374 | -1.0642 | 0.3152 |
| dt | Decision Tree Regressor | 976.2603 | 1424997.88 | 1193.0392 | -1.262 | 0.3505 |
| kr | Kernel Ridge | 1264.4727 | 2542761.74 | 1590.823 | -3.0064 | 0.5481 |
| mlp | MLP Regressor | 1483.1334 | 3311658.16 | 1811.2541 | -4.1608 | 0.6568 |
