# Supplementary material for: Perfluorinated chemicals and adolescent respiratory health: Epidemiological evidence and mechanistic insights
Source: PLoS One. 2025 Nov 14;20(11):e0336788. doi: 10.1371/journal.pone.0336788 (PMC12617853; doi:10.1371/journal.pone.0336788)
Supplement: S3 Table — (DOCX) [file pone.0336788.s012.docx]

**Perfluorinated chemicals and adolescent respiratory health: Epidemiological evidence and mechanistic insights**

Xinfeng Xu^¶^, Xinyao Jiang^¶^, Meng Zou, Jinyan Hui, Guang Huang^*^, [Qian Wu](https://pubmed.ncbi.nlm.nih.gov/?term=Wu+Q&cauthor_id=36136199)^*^

China International Cooperation Center (CCC) for Environment and Human Health and Department of Health Inspection and Quarantine, School of Public Health, Nanjing Medical University, Nanjing, China.

E-mail addresses: scottsmith@stu.njmu.edu.cn (X. Xu), jiang_xy0604@stu.njmu.edu.cn (X. Jiang), 2022121213@stu.njmu.edu.cn (M. Zou), 2024120805@stu.njmu.edu.cn (J. Hui), guanghuang@njmu.edu.cn (G. Huang), wuqian@njmu.edu.cn (Q. Wu).

^*^Corresponding authors: wuqian@njmu.edu.cn (Q. Wu); guanghuang@njmu.edu.cn (G. Huang).

^¶^Co-first authors have equal contributions to the work.

**Highlights**

- **The serum PFCs were associated with lung health among adolescents.**
- **PFOA was the dominant contributor in mixed PFC exposures.**
- **Oxidative stress may be contributed to PFC-related respiratory toxicity.**

**S3 Table. Performance of the machine learning model for regression of “FVC”**

| Abbr. | Model | MAE | MSE | RMSE | R2 | RMSLE |
| --- | --- | --- | --- | --- | --- | --- |
| ard | Automatic Relevance Determination | 766.0268 | 903197.86 | 944.3746 | 0.046 | 0.2385 |
| en | Elastic Net | 772.2342 | 910201.054 | 948.0931 | 0.0386 | 0.2392 |
| br | Bayesian Ridge | 772.0568 | 911081.363 | 948.5314 | 0.0377 | 0.2394 |
| lasso | Lasso Regression | 767.0577 | 911090.998 | 948.4988 | 0.0376 | 0.2393 |
| llar | Lasso Least Angle Regression | 767.0574 | 911090.371 | 948.4984 | 0.0376 | 0.2393 |
| ridge | Ridge Regression | 767.0608 | 911344.977 | 948.6402 | 0.0372 | 0.2393 |
| lr | Linear Regression | 767.2278 | 911553.619 | 948.7526 | 0.037 | 0.2394 |
| lar | Least Angle Regression | 767.4662 | 912284.857 | 949.1488 | 0.0362 | 0.2395 |
| huber | Huber Regressor | 762.5167 | 924366.967 | 954.5934 | 0.0257 | 0.2389 |
| omp | Orthogonal Matching Pursuit | 788.6748 | 927608.262 | 957.4703 | 0.0192 | 0.2414 |
| dummy | Dummy Regressor | 796.8588 | 953656.161 | 971.5889 | -0.0108 | 0.245 |
| tr | TheilSen Regressor | 778.2816 | 969867.666 | 978.1152 | -0.0263 | 0.2444 |
| ada | AdaBoost Regressor | 811.5788 | 969699.032 | 980.0048 | -0.0312 | 0.2521 |
| svm | Support Vector Regression | 782.4963 | 977975.327 | 982.7215 | -0.0322 | 0.2425 |
| catboost | CatBoost Regressor | 788.9353 | 991351.329 | 989.3708 | -0.0512 | 0.2501 |
| gbr | Gradient Boosting Regressor | 777.4864 | 994101.259 | 990.7018 | -0.0536 | 0.249 |
| rf | Random Forest Regressor | 794.0997 | 1006054.55 | 997.7526 | -0.0705 | 0.2513 |
| et | Extra Trees Regressor | 803.1925 | 1022124.15 | 1006.9305 | -0.0913 | 0.2531 |
| par | Passive Aggressive Regressor | 814.3302 | 1088528.81 | 1034.5176 | -0.1529 | 0.2575 |
| knn | K Neighbors Regressor | 842.6125 | 1105289.55 | 1048.2101 | -0.1909 | 0.2627 |
| lightgbm | Light Gradient Boosting Machine | 830.3208 | 1122425.42 | 1052.9111 | -0.1913 | 0.2655 |
| xgboost | Extreme Gradient Boosting | 848.0302 | 1174629.05 | 1080.0845 | -0.2603 | 0.272 |
| ransac | Random Sample Consensus | 981.4196 | 1689844.88 | 1292.9274 | -0.8192 | 0.3553 |
| dt | Decision Tree Regressor | 1119.0157 | 2032156.91 | 1422.1803 | -1.2018 | 0.3539 |
| kr | Kernel Ridge | 1445.3646 | 3363916.17 | 1815.9767 | -2.5846 | 0.554 |
| mlp | MLP Regressor | 1671.6944 | 4293709.52 | 2051.8852 | -3.5855 | 0.663 |
