# Supplementary material for: Perfluorinated chemicals and adolescent respiratory health: Epidemiological evidence and mechanistic insights
Source: PLoS One. 2025 Nov 14;20(11):e0336788. doi: 10.1371/journal.pone.0336788 (PMC12617853; doi:10.1371/journal.pone.0336788)
Supplement: S4 Table — (DOCX) [file pone.0336788.s013.docx]

**Perfluorinated chemicals and adolescent respiratory health: Epidemiological evidence and mechanistic insights**

Xinfeng Xu^¶^, Xinyao Jiang^¶^, Meng Zou, Jinyan Hui, Guang Huang^*^, [Qian Wu](https://pubmed.ncbi.nlm.nih.gov/?term=Wu+Q&cauthor_id=36136199)^*^

China International Cooperation Center (CCC) for Environment and Human Health and Department of Health Inspection and Quarantine, School of Public Health, Nanjing Medical University, Nanjing, China.

E-mail addresses: scottsmith@stu.njmu.edu.cn (X. Xu), jiang_xy0604@stu.njmu.edu.cn (X. Jiang), 2022121213@stu.njmu.edu.cn (M. Zou), 2024120805@stu.njmu.edu.cn (J. Hui), guanghuang@njmu.edu.cn (G. Huang), wuqian@njmu.edu.cn (Q. Wu).

^*^Corresponding authors: wuqian@njmu.edu.cn (Q. Wu); guanghuang@njmu.edu.cn (G. Huang).

^¶^Co-first authors have equal contributions to the work.

**Highlights**

- **The serum PFCs were associated with lung health among adolescents.**
- **PFOA was the dominant contributor in mixed PFC exposures.**
- **Oxidative stress may be contributed to PFC-related respiratory toxicity.**

**S4 Table. Performance of the machine learning model for regression of “FEV_1_/FVC”**

| Abbr. | Model | MAE | MSE | RMSE | R2 | RMSLE |
| --- | --- | --- | --- | --- | --- | --- |
| svm | Support Vector Regression | 5.2613 | 45.2746 | 6.7049 | -0.0112 | 0.079 |
| ard | Automatic Relevance Determination | 5.301 | 45.2031 | 6.7042 | -0.0119 | 0.0788 |
| lar | Least Angle Regression | 5.2988 | 45.2843 | 6.7085 | -0.0129 | 0.0788 |
| ridge | Ridge Regression | 5.2924 | 45.275 | 6.7091 | -0.0134 | 0.0788 |
| lr | Linear Regression | 5.2934 | 45.2898 | 6.71 | -0.0137 | 0.0788 |
| dummy | Dummy Regressor | 5.3097 | 45.2967 | 6.7128 | -0.015 | 0.0789 |
| br | Bayesian Ridge | 5.3099 | 45.3034 | 6.7132 | -0.0151 | 0.0789 |
| en | Elastic Net | 5.3093 | 45.3222 | 6.7143 | -0.0154 | 0.0789 |
| huber | Huber Regressor | 5.2849 | 45.4166 | 6.7183 | -0.016 | 0.079 |
| llar | Lasso Least Angle Regression | 5.312 | 45.3505 | 6.7166 | -0.0162 | 0.0789 |
| lasso | Lasso Regression | 5.312 | 45.3505 | 6.7166 | -0.0162 | 0.0789 |
| omp | Orthogonal Matching Pursuit | 5.3067 | 45.3842 | 6.7185 | -0.0168 | 0.0789 |
| ada | AdaBoost Regressor | 5.4473 | 47.2259 | 6.8588 | -0.0626 | 0.0802 |
| tr | TheilSen Regressor | 5.4659 | 48.4512 | 6.9319 | -0.0822 | 0.0815 |
| catboost | CatBoost Regressor | 5.5119 | 48.8224 | 6.9681 | -0.098 | 0.0817 |
| rf | Random Forest Regressor | 5.5785 | 49.743 | 7.0331 | -0.1164 | 0.0825 |
| gbr | Gradient Boosting Regressor | 5.5138 | 49.8196 | 7.0378 | -0.1178 | 0.0826 |
| et | Extra Trees Regressor | 5.5949 | 50.4702 | 7.0873 | -0.1338 | 0.0832 |
| knn | K Neighbors Regressor | 5.7827 | 53.6255 | 7.3067 | -0.2088 | 0.0856 |
| lightgbm | Light Gradient Boosting Machine | 5.8767 | 55.3595 | 7.4298 | -0.2495 | 0.0871 |
| xgboost | Extreme Gradient Boosting | 6.1257 | 59.7392 | 7.7089 | -0.3418 | 0.0905 |
| ransac | Random Sample Consensus | 6.912 | 82.387 | 9.0149 | -0.8422 | 0.1071 |
| mlp | MLP Regressor | 7.4117 | 89.2306 | 9.3581 | -1.015 | 0.1066 |
| dt | Decision Tree Regressor | 7.5304 | 91.402 | 9.5214 | -1.0607 | 0.1124 |
| par | Passive Aggressive Regressor | 7.555 | 98.3121 | 9.5129 | -1.1641 | 0.1153 |
| kr | Kernel Ridge | 31.4745 | 1505.6003 | 38.622 | -33.3192 | 0.558 |
