# Supplementary material for: Perfluorinated chemicals and adolescent respiratory health: Epidemiological evidence and mechanistic insights
Source: PLoS One. 2025 Nov 14;20(11):e0336788. doi: 10.1371/journal.pone.0336788 (PMC12617853; doi:10.1371/journal.pone.0336788)
Supplement: S5 Table — (DOCX) [file pone.0336788.s014.docx]

**Perfluorinated chemicals and adolescent respiratory health: Epidemiological evidence and mechanistic insights**

Xinfeng Xu^¶^, Xinyao Jiang^¶^, Meng Zou, Jinyan Hui, Guang Huang^*^, [Qian Wu](https://pubmed.ncbi.nlm.nih.gov/?term=Wu+Q&cauthor_id=36136199)^*^

China International Cooperation Center (CCC) for Environment and Human Health and Department of Health Inspection and Quarantine, School of Public Health, Nanjing Medical University, Nanjing, China.

E-mail addresses: scottsmith@stu.njmu.edu.cn (X. Xu), jiang_xy0604@stu.njmu.edu.cn (X. Jiang), 2022121213@stu.njmu.edu.cn (M. Zou), 2024120805@stu.njmu.edu.cn (J. Hui), guanghuang@njmu.edu.cn (G. Huang), wuqian@njmu.edu.cn (Q. Wu).

^*^Corresponding authors: wuqian@njmu.edu.cn (Q. Wu); guanghuang@njmu.edu.cn (G. Huang).

^¶^Co-first authors have equal contributions to the work.

**Highlights**

- **The serum PFCs were associated with lung health among adolescents.**
- **PFOA was the dominant contributor in mixed PFC exposures.**
- **Oxidative stress may be contributed to PFC-related respiratory toxicity.**

**S5 Table. Performance of the machine learning model for classification of “Wheeze”**

| Abbr. | Model | Accuracy | AUC | Recall | Prec. | F1 | Kappa |
| --- | --- | --- | --- | --- | --- | --- | --- |
| lr | Logistic Regression | 0.8932 | 0.5446 | 0 | 0 | 0 | 0 |
| lda | Linear Discriminant Analysis | 0.8932 | 0.5326 | 0 | 0 | 0 | 0 |
| rbfsvm | SVM - Radial Kernel | 0.8932 | 0.5537 | 0 | 0 | 0 | 0 |
| dummy | Dummy Classifier | 0.8932 | 0.5 | 0 | 0 | 0 | 0 |
| ridge | Ridge Classifier | 0.8932 | 0.5321 | 0 | 0 | 0 | 0 |
| mlp | MLP Classifier | 0.8931 | 0.5388 | 0.0125 | 0.1 | 0.0222 | 0.0175 |
| et | Extra Trees Classifier | 0.8917 | 0.5847 | 0 | 0 | 0 | -0.0026 |
| rf | Random Forest Classifier | 0.8887 | 0.566 | 0 | 0 | 0 | -0.0074 |
| gpc | Gaussian Process Classifier | 0.8858 | 0.459 | 0 | 0 | 0 | -0.0119 |
| knn | K Neighbors Classifier | 0.8858 | 0.5605 | 0.0268 | 0.15 | 0.0444 | 0.0258 |
| gbc | Gradient Boosting Classifier | 0.8844 | 0.5756 | 0.0286 | 0.2 | 0.05 | 0.0258 |
| catboost | CatBoost Classifier | 0.8815 | 0.6004 | 0.0411 | 0.1333 | 0.0622 | 0.0318 |
| lightgbm | Light Gradient Boosting Machine | 0.8814 | 0.5421 | 0.0286 | 0.2 | 0.05 | 0.0211 |
| nb | Naive Bayes | 0.877 | 0.5745 | 0.0518 | 0.1417 | 0.0748 | 0.0365 |
| qda | Quadratic Discriminant Analysis | 0.8756 | 0.5775 | 0.0518 | 0.125 | 0.073 | 0.0331 |
| ada | Ada Boost Classifier | 0.8755 | 0.574 | 0.0268 | 0.0833 | 0.04 | 0.005 |
| xgboost | Extreme Gradient Boosting | 0.8653 | 0.5477 | 0.0411 | 0.1167 | 0.0604 | 0.0083 |
| dt | Decision Tree Classifier | 0.7907 | 0.5041 | 0.1393 | 0.1066 | 0.1199 | 0.0042 |
| svm | SVM - Linear Kernel | 0.749 | 0.528 | 0.1429 | 0.0408 | 0.0462 | -0.0062 |
