# Supplementary material for: Perfluorinated chemicals and adolescent respiratory health: Epidemiological evidence and mechanistic insights
Source: PLoS One. 2025 Nov 14;20(11):e0336788. doi: 10.1371/journal.pone.0336788 (PMC12617853; doi:10.1371/journal.pone.0336788)
Supplement: S6 Table — (DOCX) [file pone.0336788.s015.docx]

**Perfluorinated chemicals and adolescent respiratory health: Epidemiological evidence and mechanistic insights**

Xinfeng Xu^¶^, Xinyao Jiang^¶^, Meng Zou, Jinyan Hui, Guang Huang^*^, [Qian Wu](https://pubmed.ncbi.nlm.nih.gov/?term=Wu+Q&cauthor_id=36136199)^*^

China International Cooperation Center (CCC) for Environment and Human Health and Department of Health Inspection and Quarantine, School of Public Health, Nanjing Medical University, Nanjing, China.

E-mail addresses: scottsmith@stu.njmu.edu.cn (X. Xu), jiang_xy0604@stu.njmu.edu.cn (X. Jiang), 2022121213@stu.njmu.edu.cn (M. Zou), 2024120805@stu.njmu.edu.cn (J. Hui), guanghuang@njmu.edu.cn (G. Huang), wuqian@njmu.edu.cn (Q. Wu).

^*^Corresponding authors: wuqian@njmu.edu.cn (Q. Wu); guanghuang@njmu.edu.cn (G. Huang).

^¶^Co-first authors have equal contributions to the work.

**Highlights**

- **The serum PFCs were associated with lung health among adolescents.**
- **PFOA was the dominant contributor in mixed PFC exposures.**
- **Oxidative stress may be contributed to PFC-related respiratory toxicity.**

**S6 Table. Performance of the machine learning model for classification of “Asthma”**

| Abbr. | Model | Accuracy | AUC | Recall | Prec. | F1 | Kappa |
| --- | --- | --- | --- | --- | --- | --- | --- |
| lr | Logistic Regression | 0.8038 | 0.4445 | 0 | 0 | 0 | 0 |
| ridge | Ridge Classifier | 0.8038 | 0.4434 | 0 | 0 | 0 | 0 |
| dummy | Dummy Classifier | 0.8038 | 0.5 | 0 | 0 | 0 | 0 |
| lda | Linear Discriminant Analysis | 0.8038 | 0.4425 | 0 | 0 | 0 | 0 |
| rbfsvm | SVM - Radial Kernel | 0.8024 | 0.4925 | 0 | 0 | 0 | -0.0028 |
| mlp | MLP Classifier | 0.8024 | 0.4222 | 0 | 0 | 0 | -0.0028 |
| ada | Ada Boost Classifier | 0.7936 | 0.5206 | 0.0225 | 0.1333 | 0.0383 | 0.0056 |
| et | Extra Trees Classifier | 0.7906 | 0.4518 | 0.0148 | 0.15 | 0.0267 | -0.0072 |
| rf | Random Forest Classifier | 0.7877 | 0.4231 | 0.0071 | 0.05 | 0.0125 | -0.0219 |
| gpc | Gaussian Process Classifier | 0.7848 | 0.4734 | 0.0445 | 0.1867 | 0.0707 | 0.0131 |
| gbc | Gradient Boosting Classifier | 0.7832 | 0.4796 | 0.0291 | 0.1833 | 0.0481 | -0.006 |
| catboost | CatBoost Classifier | 0.7745 | 0.505 | 0.0533 | 0.245 | 0.0833 | 0.0064 |
| knn | K Neighbors Classifier | 0.773 | 0.5055 | 0.0813 | 0.28 | 0.1207 | 0.0329 |
| nb | Naive Bayes | 0.773 | 0.4642 | 0.0379 | 0.1367 | 0.0589 | -0.013 |
| qda | Quadratic Discriminant Analysis | 0.7716 | 0.4721 | 0.0231 | 0.075 | 0.0353 | -0.0319 |
| lightgbm | Light Gradient Boosting Machine | 0.7525 | 0.452 | 0.0736 | 0.1942 | 0.1025 | -0.0096 |
| xgboost | Extreme Gradient Boosting | 0.7319 | 0.4668 | 0.0665 | 0.1193 | 0.0837 | -0.0484 |
| svm | SVM - Linear Kernel | 0.7128 | 0.4958 | 0.1302 | 0.0705 | 0.0699 | -0.0185 |
| dt | Decision Tree Classifier | 0.6296 | 0.4417 | 0.133 | 0.114 | 0.121 | -0.1091 |
