# Supplementary material for: Perfluorinated chemicals and adolescent respiratory health: Epidemiological evidence and mechanistic insights
Source: PLoS One. 2025 Nov 14;20(11):e0336788. doi: 10.1371/journal.pone.0336788 (PMC12617853; doi:10.1371/journal.pone.0336788)
Supplement: S7 Table — (DOCX) [file pone.0336788.s016.docx]

**Perfluorinated chemicals and adolescent respiratory health: Epidemiological evidence and mechanistic insights**

Xinfeng Xu^¶^, Xinyao Jiang^¶^, Meng Zou, Jinyan Hui, Guang Huang^*^, [Qian Wu](https://pubmed.ncbi.nlm.nih.gov/?term=Wu+Q&cauthor_id=36136199)^*^

China International Cooperation Center (CCC) for Environment and Human Health and Department of Health Inspection and Quarantine, School of Public Health, Nanjing Medical University, Nanjing, China.

E-mail addresses: scottsmith@stu.njmu.edu.cn (X. Xu), jiang_xy0604@stu.njmu.edu.cn (X. Jiang), 2022121213@stu.njmu.edu.cn (M. Zou), 2024120805@stu.njmu.edu.cn (J. Hui), guanghuang@njmu.edu.cn (G. Huang), wuqian@njmu.edu.cn (Q. Wu).

^*^Corresponding authors: wuqian@njmu.edu.cn (Q. Wu); guanghuang@njmu.edu.cn (G. Huang).

^¶^Co-first authors have equal contributions to the work.

**Highlights**

- **The serum PFCs were associated with lung health among adolescents.**
- **PFOA was the dominant contributor in mixed PFC exposures.**
- **Oxidative stress may be contributed to PFC-related respiratory toxicity.**

**S7 Table. Association between serum PFCs and immune indices and oxidative stress index**

|  | **SII** | | **NLR** | | **SIRI** | | **gamma-glutamyl transferase** | | **total bilirubin** | |
| --- | --- | --- | --- | --- | --- | --- | --- | --- | --- | --- |
|  | Adjusted β  (95% CI) | *p* | Adjusted β  (95% CI) | *p* | Adjusted β  (95% CI) | *p* | Adjusted β  (95% CI) | *p* | Adjusted β  (95% CI) | *p* |
| **PFOA** | -3.3 (-14.59,7.99) | 0.566 | -0.04 (-0.07,0) | 0.053 | -0.01 (-0.04,0.02 | 0.618 | 0.1 (-0.23,0.43) | 0.556 | 0.02 (0,0.03) | 0.012* |
| **PFNA** | -21.21 (-45.92,3.5) | 0.092 | -0.05 (-0.14,0.03) | 0.193 | -0.06 (-0.13,0.01) | 0.112 | -0.01 (-0.73,0.72) | 0.986 | 0 (-0.03,0.02) | 0.813 |
| **PFDE** | -59.36 (-166.53,47.82) | 0.277 | -0.22 (-0.58,0.13) | 0.213 | -0.3 (-0.61,0) | 0.051 | -1.9 (-5.03,1.23) | 0.234 | 0.01 (-0.11,0.14) | 0.819 |
| **PFHS** | 3.41 (-0.47,7.3) | 0.085 | 0 (-0.01,0.02) | 0.546 | 0.01 (0,0.02) | 0.199 | 0.03 (-0.08,0.14) | 0.608 | 0 (0,0.01) | 0.241 |
| **PFOS** | 1.54 (-1.27,4.35) | 0.282 | 0 (-0.01,0.01) | 0.668 | 0 (-0.01,0.01) | 0.672 | 0.06 (-0.02,0.14) | 0.151 | 0 (0,0.01) | 0.059 |
| **MPAH** | 28.47 (-13.08,70.01) | 0.179 | 0.05 (-0.09,0.19) | 0.471 | 0.07 (-0.05,0.19) | 0.251 | -0.29 (-1.5,0.92) | 0.639 | -0.01 (-0.06,0.03) | 0.556 |
