# Supplementary material for: Perfluorinated chemicals and adolescent respiratory health: Epidemiological evidence and mechanistic insights
Source: PLoS One. 2025 Nov 14;20(11):e0336788. doi: 10.1371/journal.pone.0336788 (PMC12617853; doi:10.1371/journal.pone.0336788)
Supplement: S8 Table — (DOCX) [file pone.0336788.s017.docx]

**Perfluorinated chemicals and adolescent respiratory health: Epidemiological evidence and mechanistic insights**

Xinfeng Xu^¶^, Xinyao Jiang^¶^, Meng Zou, Jinyan Hui, Guang Huang^*^, [Qian Wu](https://pubmed.ncbi.nlm.nih.gov/?term=Wu+Q&cauthor_id=36136199)^*^

China International Cooperation Center (CCC) for Environment and Human Health and Department of Health Inspection and Quarantine, School of Public Health, Nanjing Medical University, Nanjing, China.

E-mail addresses: scottsmith@stu.njmu.edu.cn (X. Xu), jiang_xy0604@stu.njmu.edu.cn (X. Jiang), 2022121213@stu.njmu.edu.cn (M. Zou), 2024120805@stu.njmu.edu.cn (J. Hui), guanghuang@njmu.edu.cn (G. Huang), wuqian@njmu.edu.cn (Q. Wu).

^*^Corresponding authors: wuqian@njmu.edu.cn (Q. Wu); guanghuang@njmu.edu.cn (G. Huang).

^¶^Co-first authors have equal contributions to the work.

**Highlights**

- **The serum PFCs were associated with lung health among adolescents.**
- **PFOA was the dominant contributor in mixed PFC exposures.**
- **Oxidative stress may be contributed to PFC-related respiratory toxicity.**

**S8 Table. Association between immune indices and oxidative stress index and lung health parameters**

|  | **FEV_1_** | | **FVC** | | **FEV_1_/FVC** | | **Wheeze** | | **Asthma** | |
| --- | --- | --- | --- | --- | --- | --- | --- | --- | --- | --- |
|  | Adjusted β  (95% CI) | *p* | Adjusted β  (95% CI) | *p* | Adjusted β  (95% CI) | *p* | Adjusted β  (95% CI) | *p* | Adjusted β  (95% CI) | *p* |
| SII | 0.13 (-0.01,0.27) | 0.08 | 0.11 (-0.05,0.27) | 0.176 | 0 (0,0) | 0.298 | 0 (0,0) | 0.988 | 0 (0,0) | 0.514 |
| NLR | 72.67 (30.15,115.19) | <0.001*** | 72.11 (22.96,121.26) | 0.004** | 0.32 (-0.14,0.78) | 0.174 | 0 (-0.02,0.02) | 0.998 | -0.01 (-0.04,0.02) | 0.388 |
| SIRI | 72.62 (23.64,121.59) | 0.004** | 80.51 (23.97,137.06) | 0.005** | 0.12 (-0.41,0.65) | 0.652 | -0.01 (-0.03,0.02) | 0.576 | -0.02 (-0.05,0.01) | 0.169 |
| GGT | -5.13 (-9.94,-0.33) | 0.036* | -6.27 (-11.81,-0.73) | 0.027* | 0.01 (-0.04,0.07) | 0.588 | 0 (0,0) | 0.179 | 0 (-0.01,0) | 0.211 |
| TOTALBR | 231.87 (109.53,354.22) | <0.001*** | 310.44 (169.58,451.3) | <0.001*** | -0.58 (-1.91,0.74) | 0.386 | -0.01 (-0.07,0.05) | 0.728 | -0.05 (-0.13,0.03) | 0.25 |
