# Supplementary material for: Perfluorinated chemicals and adolescent respiratory health: Epidemiological evidence and mechanistic insights
Source: PLoS One. 2025 Nov 14;20(11):e0336788. doi: 10.1371/journal.pone.0336788 (PMC12617853; doi:10.1371/journal.pone.0336788)
Supplement: S9 Table — (DOCX) [file pone.0336788.s018.docx]

**Perfluorinated chemicals and adolescent respiratory health: Epidemiological evidence and mechanistic insights**

Xinfeng Xu^¶^, Xinyao Jiang^¶^, Meng Zou, Jinyan Hui, Guang Huang^*^, [Qian Wu](https://pubmed.ncbi.nlm.nih.gov/?term=Wu+Q&cauthor_id=36136199)^*^

China International Cooperation Center (CCC) for Environment and Human Health and Department of Health Inspection and Quarantine, School of Public Health, Nanjing Medical University, Nanjing, China.

E-mail addresses: scottsmith@stu.njmu.edu.cn (X. Xu), jiang_xy0604@stu.njmu.edu.cn (X. Jiang), 2022121213@stu.njmu.edu.cn (M. Zou), 2024120805@stu.njmu.edu.cn (J. Hui), guanghuang@njmu.edu.cn (G. Huang), wuqian@njmu.edu.cn (Q. Wu).

^*^Corresponding authors: wuqian@njmu.edu.cn (Q. Wu); guanghuang@njmu.edu.cn (G. Huang).

^¶^Co-first authors have equal contributions to the work.

**Highlights**

- **The serum PFCs were associated with lung health among adolescents.**
- **PFOA was the dominant contributor in mixed PFC exposures.**
- **Oxidative stress may be contributed to PFC-related respiratory toxicity.**

**S9 Table. Demographic characteristics of participants aged 12‐19 years with available data in the NHANES 2013‐2018 cycles (N = 3352)**

|  | All | Male | Female |
| --- | --- | --- | --- |
|  | (N=3352) | (N=1715) | (N=1637) |
| **Age (years),**  **Mean ± SD** | 15.41 ± 2.24 | 15.41 ± 2.24 | 15.46 ± 2.24 |
| **Oxidative Stress, Mean ± SD** | | | |
| Total Bilirubin,  (µmol/L) | 0.55 ± 0.34 | 0.61 ± 0.37 | 0.49 ± 0.30 |
| Gamma glutamyl transferase, (U/L) | 14.70 ± 10.15 | 16.57 ± 11.14 | 12.75 ± 8.58 |
| **Inflammatory Markers, Mean ± SD** | | | |
| NLR | 1.78 ± 0.98 | 1.70 ± 1.02 | 1.86 ± 0.94 |
| SII | 456.69 ± 270.56 | 416.12 ± 260.07 | 499.20 ± 274.85 |
| SIRI | 1.05 ± 0.85 | 1.04 ± 0.97 | 1.07 ± 0.70 |
| **Serum PFAS level (ng/mL), Mean ± SD** | | | |
| PFOA | 0.49 ± 0.85 | 0.57 ± 0.97 | 0.41 ± 0.70 |
| PFNA | 0.23 ± 0.37 | 0.26 ± 0.42 | 0.20 ± 0.30 |
| PFDE | 0.10 ± 0.11 | 0.10 ± 0.11 | 0.10 ± 0.10 |
| PFHS | 0.47 ± 1.21 | 0.58 ± 1.49 | 0.35 ± 0.79 |
| PFOS | 1.10 ± 2.28 | 1.32 ± 2.72 | 0.87 ± 1.68 |
| MPAH | 1.00 ± 0.13 | 1.11 ± 0.14 | 0.09 ± 0.12 |
| PFUA | 0.08 ± 0.06 | 0.08 ± 0.05 | 0.08 ± 0.07 |
| **Asthma (%)** | | | |
| Yes‐1 | 19.69 | 20.58 | 18.75 |
| No‐2 | 80.31 | 79.42 | 81.25 |

Note: NLR, Neutrophil to lymphocyte ratio; SII, Systemic immune inflammation index; SIRI, Systemic inflammatory response index.
